# Supplementary material for: Plasmodium falciparum Calcium-Dependent Protein Kinase 4 is Critical for Male Gametogenesis and Transmission to the Mosquito Vector
Source: mBio. 2021 Nov 2;12(6):e02575-21. doi: 10.1128/mBio.02575-21 (PMC8561384; doi:10.1128/mBio.02575-21)
Supplement: DATA SET S3 [file mbio.02575-21-sd003.pdf]

PfCDPK1

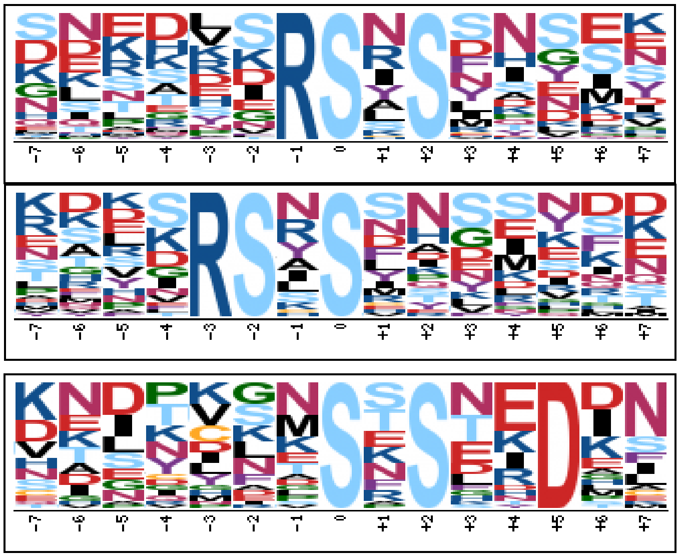

PfCDPK4

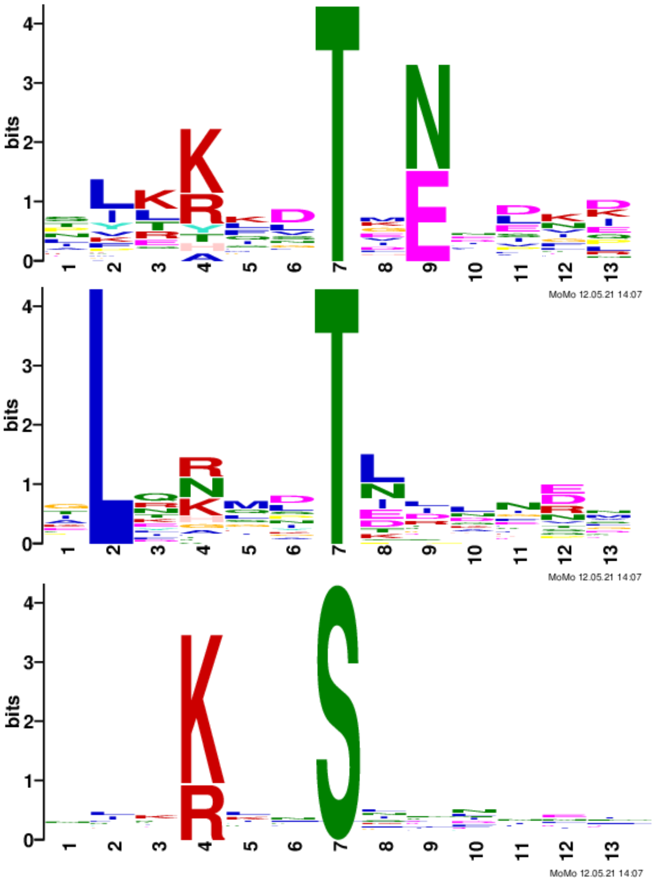

PfCDPK5

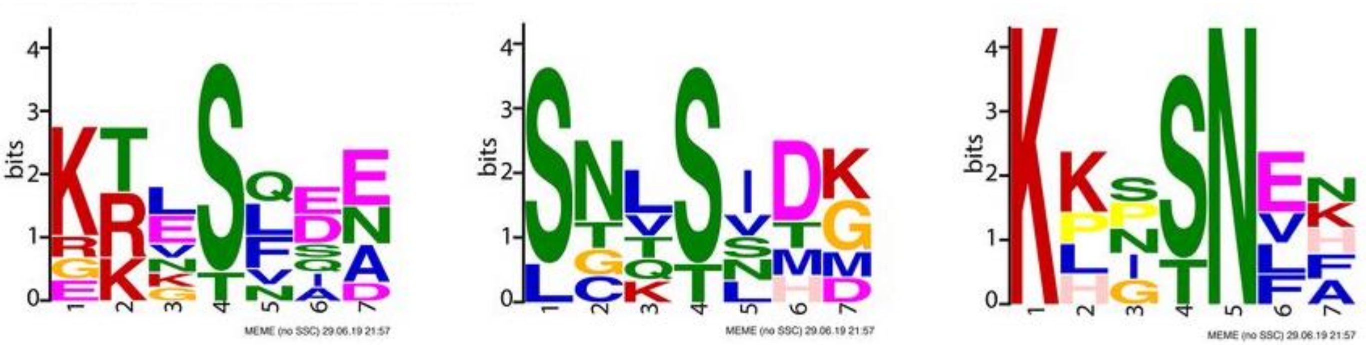

Supplementary Data S3. Comparison of motif enrichment for putative substrates of *P. falciparum* CDPK1, CDPK4, and CDPK5. PfCDPK1 is from Kumar *et al.* 2017 (PMID: 28680058) Fig. 1d. PfCDPK4 is from this manuscript, Fig. 6B. PfCDPK5 is from Blomqvist *et al.* 2020 (PMID: 31915223) Fig. 1D.
