# Supplementary material for: Plasmodium falciparum Calcium-Dependent Protein Kinase 4 is Critical for Male Gametogenesis and Transmission to the Mosquito Vector
Source: mBio. 2021 Nov 2;12(6):e02575-21. doi: 10.1128/mBio.02575-21 (PMC8561384; doi:10.1128/mBio.02575-21)
Supplement: DATA SET S5 [file mbio.02575-21-sd005.pdf]

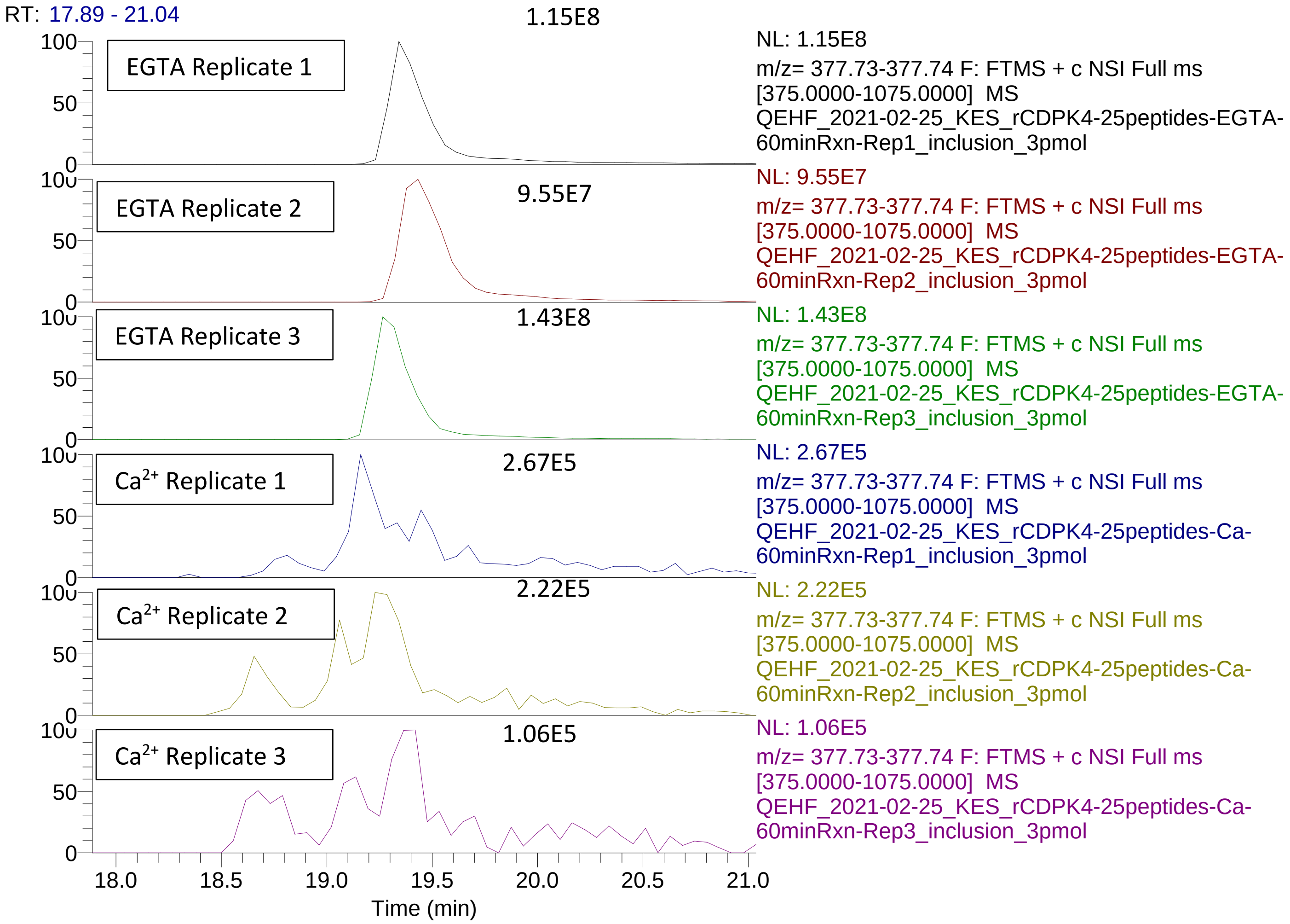

Extracted ion chromatograms for 377.74 m/z (charge +4). Peaks are labeled with peak intensity. This peptide was confidently identified in all runs as the unmodified peptide syntide 2, PLARTLSVAGLPGKK. PfCDPK4 is inactive in the presence of the calcium chelator EGTA and active in the presence of Ca^{2+}. In the replicates with active PfCDPK4, the peptide abundance was depleted an average of 99.8%, indicating that the peptide was phosphorylated.

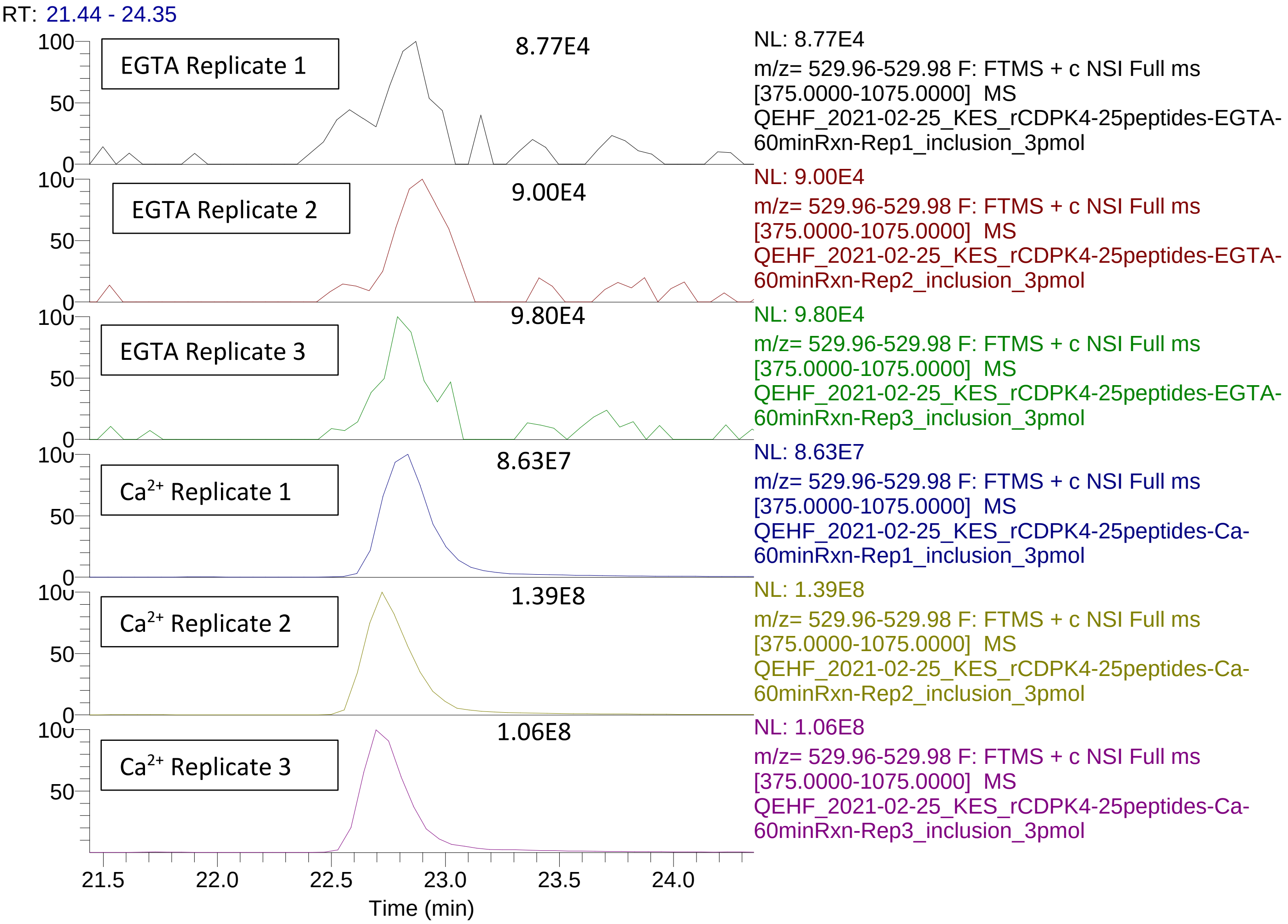

Extracted ion chromatograms for 529.97 m/z (charge +3). Peaks are labeled with peak intensity. This peptide was confidently identified in all runs as the peptide syntide 2 phosphopeptpide, PLARTLS[+79.97]VAGLPGKK. PfCDPK4 is inactive in the presence of the calcium chelator EGTA and active in the presence of Ca^{2+}. Phosphopeptide abundance in the presence of EGTA was near background levels, indicating near complete inactivation of the kinase. By contrast, the presence of Ca^{2+} was in the kinase buffer led to near complete conversion of the peptide to phosphopeptide.

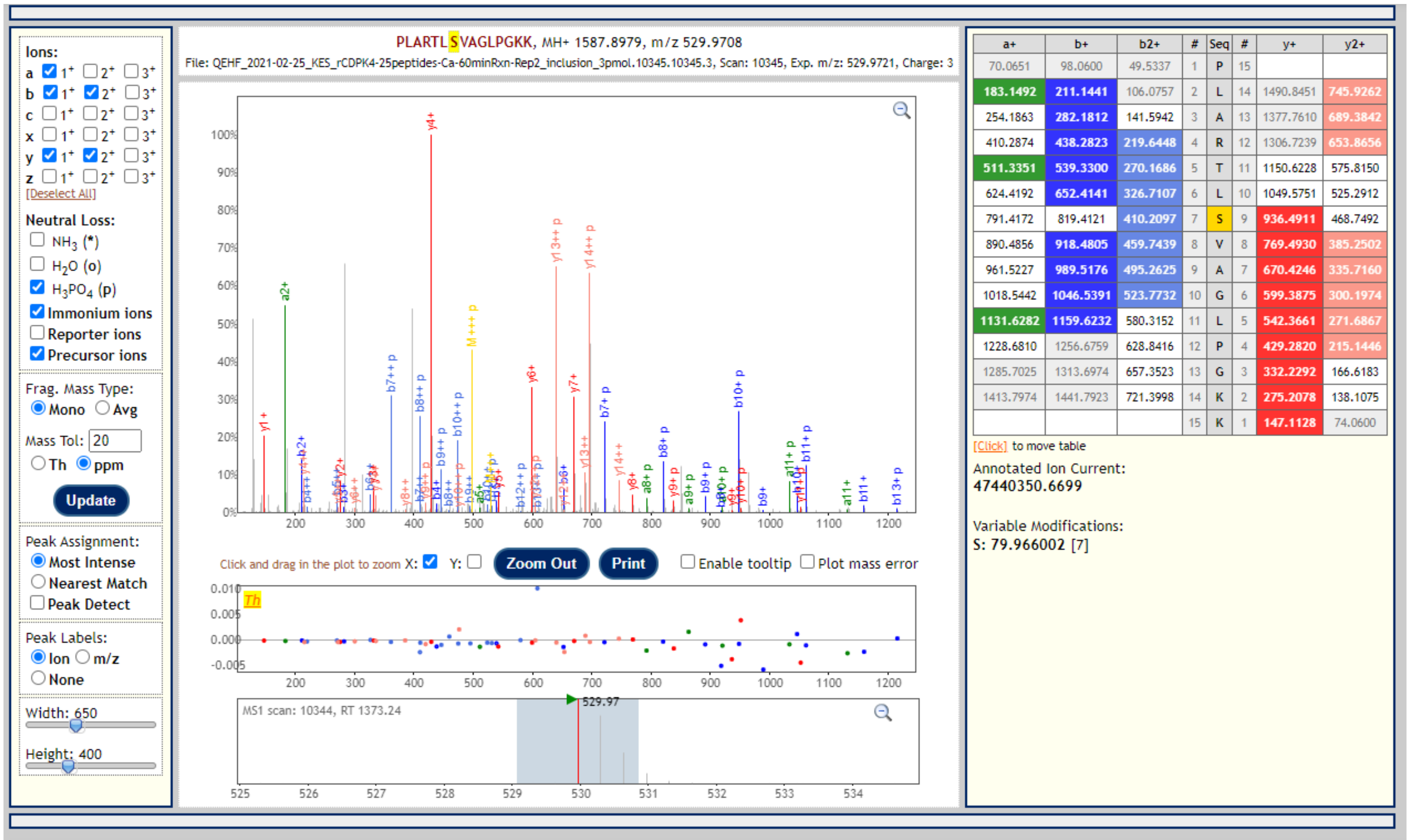

MS2 spectrum from the apex of the 529.97 m/z (3+) peak identifying the syntide 2 phosphopeptide Ca^{2+} replicate 1.
PeptideProphet probability = 1.000
PTMProphet localization scores = PLART(0.012)LS(0.988)VAGLPGKK, confidently localizing the phosphosite.

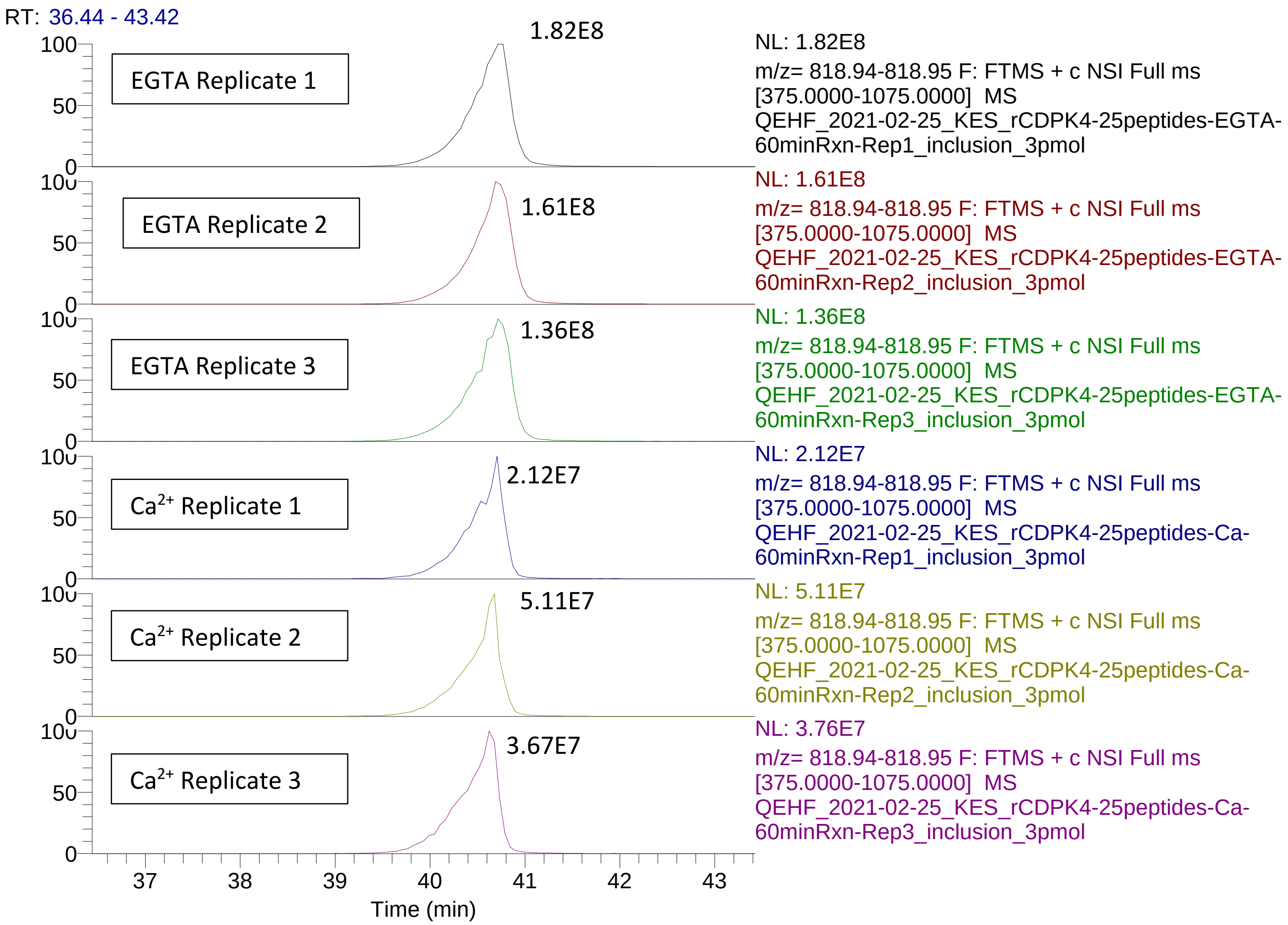

Extracted ion chromatograms for 818.95 m/z (charge +2). Peaks are labeled with peak intensity. This peptide was confidently identified in all runs as the unmodified peptide SLPYEKALSSTISQI from the uncharacterized protein PF3D7_0417600. PfCDPK4 is inactive in the presence of the calcium chelator EGTA and active in the presence of Ca^{2+}. In the replicates with active PfCDPK4, the peptide abundance was depleted an average of 77% (85% for the +3 ion), indicating that the peptide was phosphorylated.

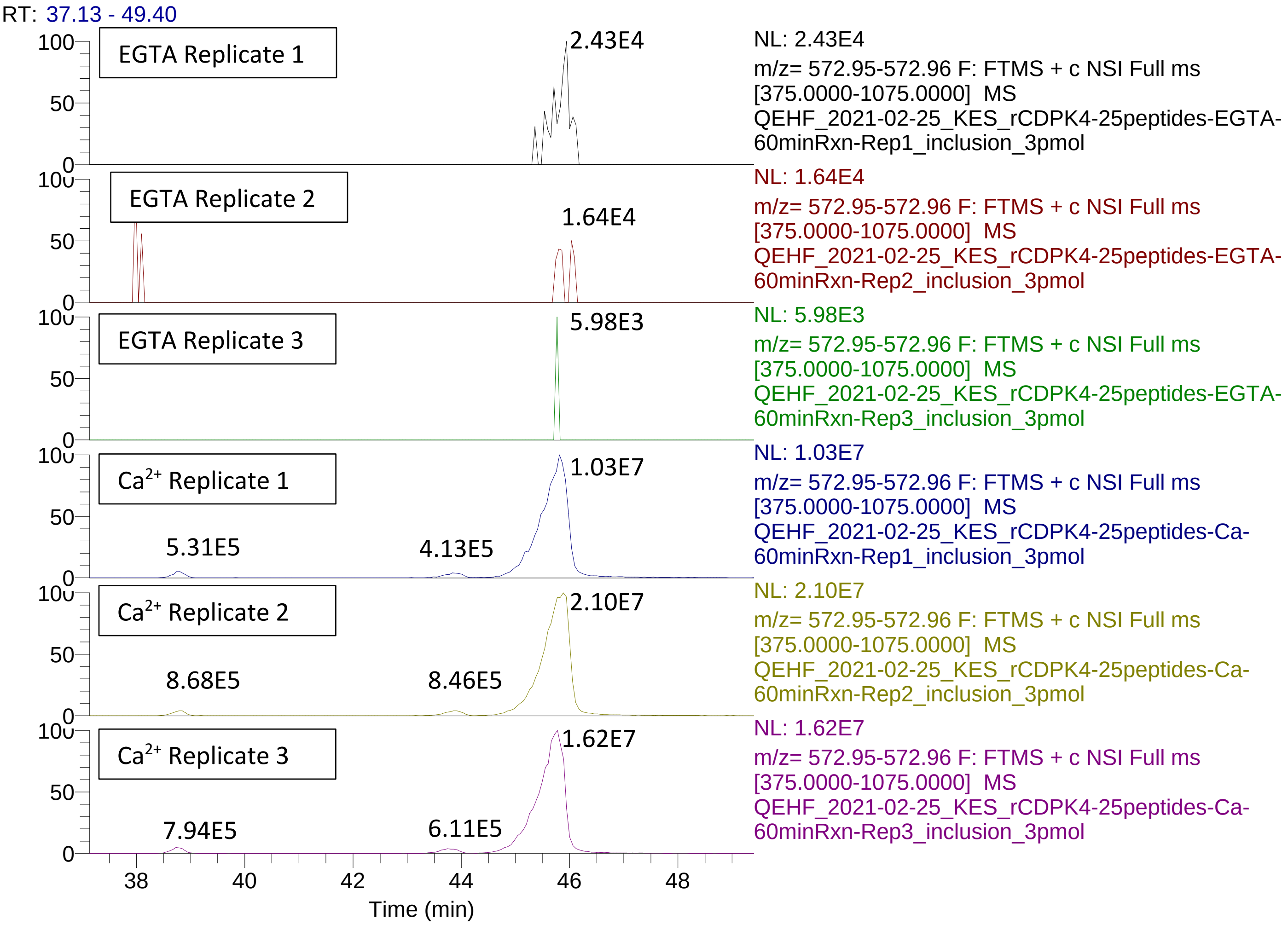

Extracted ion chromatograms for 572.95 m/z (charge +3), which matches the mass of the singly phosphorylated peptide SLPYEKALSSTISQI from the uncharacterized protein PF3D7_0417600. PfCDPK4 is inactive in the presence of the calcium chelator EGTA and active in the presence of Ca^{2+}. Peaks are labeled with peak intensity. In the replicates with active PfCDPK4, there are three isobaric species present at distinct retention times, with the major species at retention time 45.8 min. These likely represent three positional isomers of the phosphopeptide. The MS2 spectra from this species could not confidently determine the peptide sequence, nor could the phosphosite be confidently localized (see below). However, the fact that the unmodified peptide was depleted by over 77% in the presence of activated PfCDPK4 concomitant with appearance of a peptide matching the mass of the phosphopeptide supports the claim that the peptide was phosphorylated by PfCDPK4.

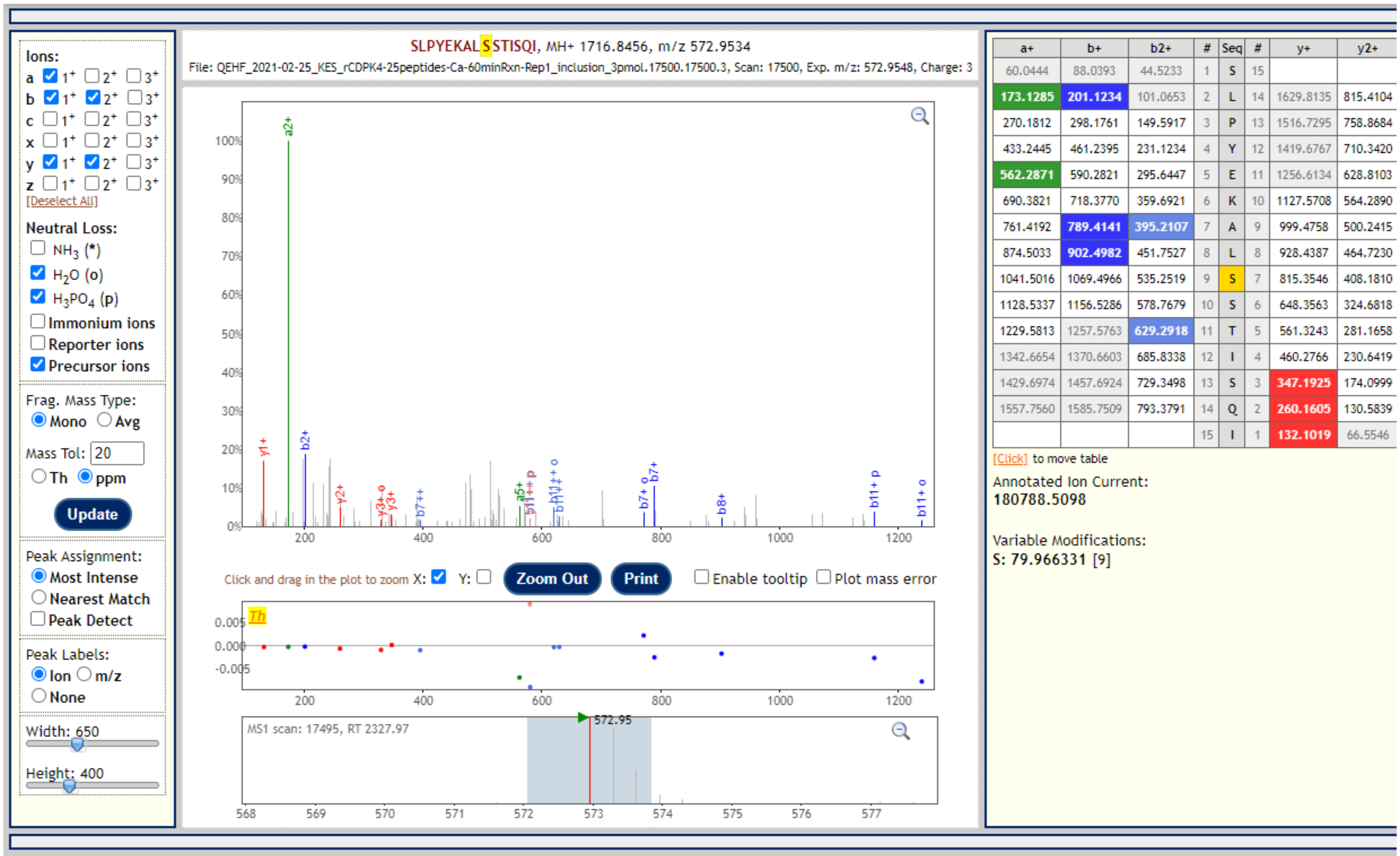

MS2 spectrum from the apex of the 572.95 m/z (3+) peak at 38.7 min in Ca^{2+} replicate 1.

PeptideProphet probability = 0.7096, below the cut-off for identifying spectra.

There are insufficient peaks to localize the phosphosite.

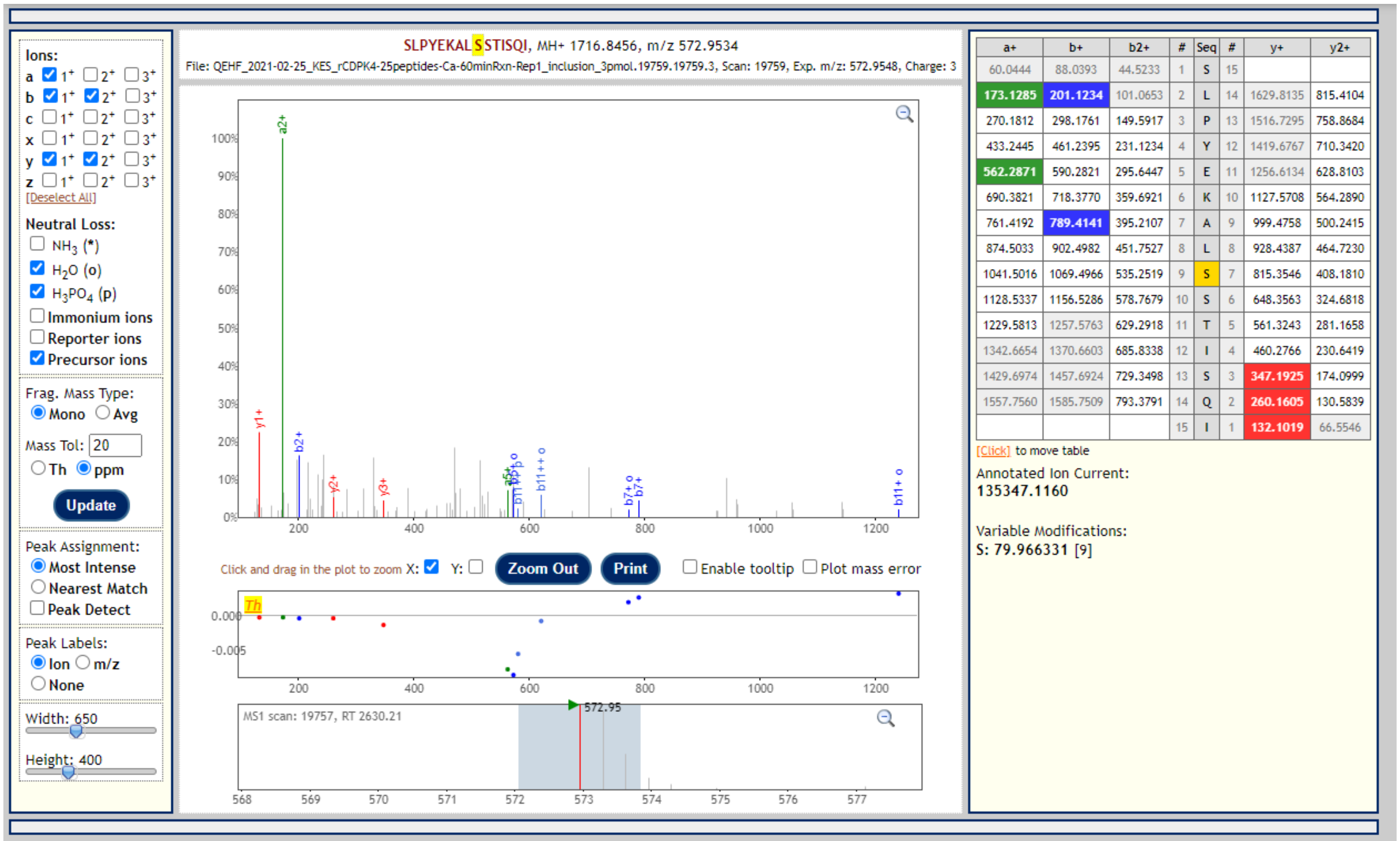

a+	b+	b2+	#	Seq	#	y+	y2+
60.0444	88.0393	44.5233	1	S	15		
173.1285	201.1234	101.0653	2	L	14	1629.8135	815.4104
270.1812	298.1761	149.5917	3	P	13	1516.7295	758.8684
433.2445	461.2395	231.1234	4	Y	12	1419.6767	710.3420
562.2871	590.2821	295.6447	5	E	11	1256.6134	628.8103
690.3821	718.3770	359.6921	6	K	10	1127.5708	564.2890
761.4192	789.4141	395.2107	7	A	9	999.4758	500.2415
874.5033	902.4982	451.7527	8	L	8	928.4387	464.7230
1041.5016	1069.4966	535.2519	9	S	7	815.3546	408.1810
1128.5337	1156.5286	578.7679	10	S	6	648.3563	324.6818
1229.5813	1257.5763	629.2918	11	T	5	561.3243	281.1658
1342.6654	1370.6603	685.8338	12	I	4	460.2766	230.6419
1429.6974	1457.6924	729.3498	13	S	3	347.1925	174.0999
1557.7560	1585.7509	793.3791	14	Q	2	260.1605	130.5839
			15	I	1	132.1019	66.5546

[Click] to move table

Annotated Ion Current:
135347.1160

Variable Modifications:
S: 79.966331 [9]

MS2 spectrum from apex of 572.95 m/z (3+) peak at 43.8 min in Ca^{2+} replicate 1.
PeptideProphet probability = 0.4826, below the cutoff for identifying spectra.
There are insufficient peaks to localize the phosphosite.

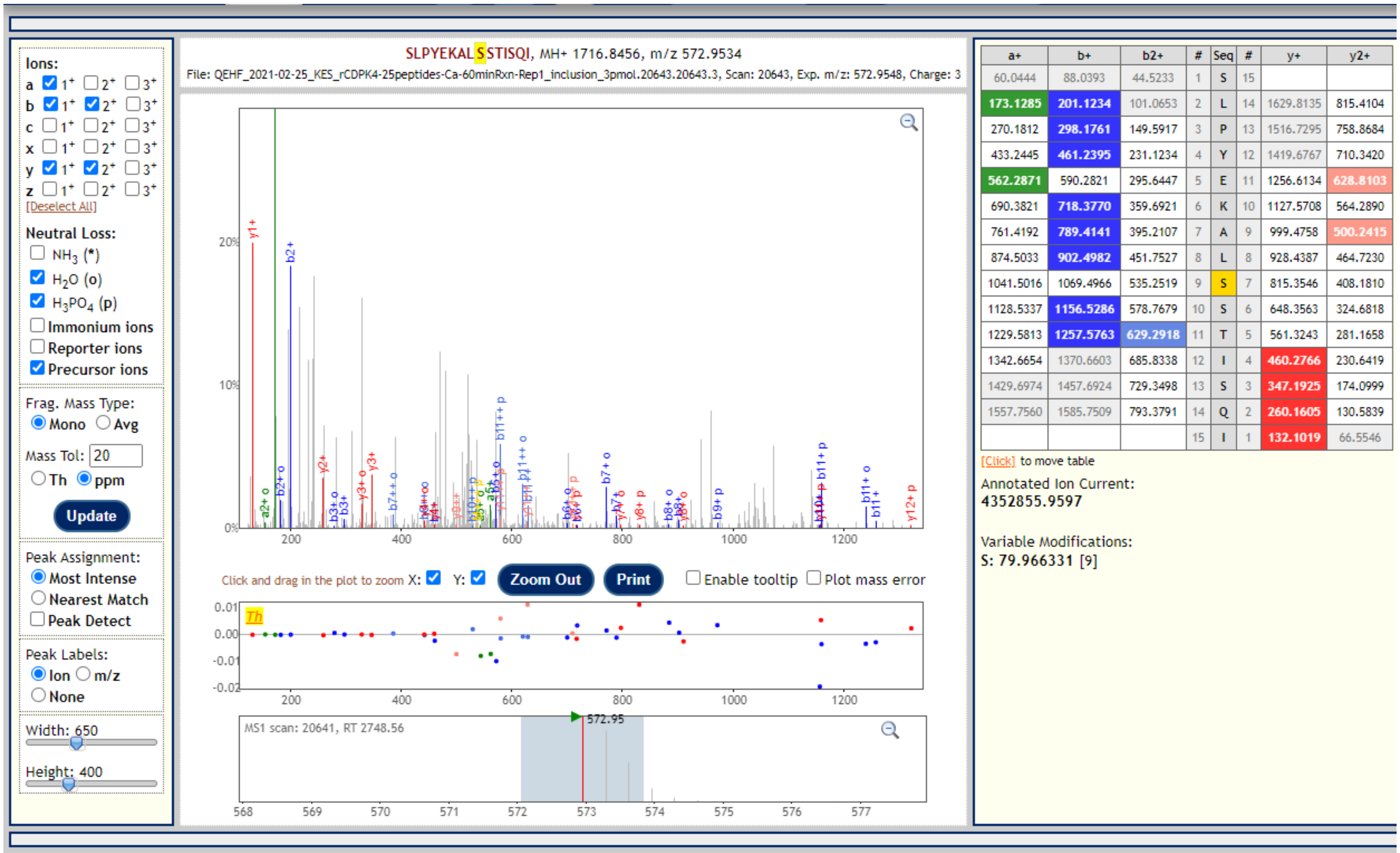

a+	b+	b2+	#	Seq	#	y+	y2+
60.0444	88.0393	44.5233	1	S	15		
173.1285	201.1234	101.0653	2	L	14	1629.8135	815.4104
270.1812	298.1761	149.5917	3	P	13	1516.7295	758.8684
433.2445	461.2395	231.1234	4	Y	12	1419.6767	710.3420
562.2871	590.2821	295.6447	5	E	11	1256.6134	628.8103
690.3821	718.3770	359.6921	6	K	10	1127.5708	564.2890
761.4192	789.4141	395.2107	7	A	9	999.4758	500.2415
874.5033	902.4982	451.7527	8	L	8	928.4387	464.7230
1041.5016	1069.4966	535.2519	9	S	7	815.3546	408.1810
1128.5337	1156.5286	578.7679	10	S	6	648.3563	324.6818
1229.5813	1257.5763	629.2918	11	T	5	561.3243	281.1658
1342.6654	1370.6603	685.8338	12	I	4	460.2766	230.6419
1429.6974	1457.6924	729.3498	13	S	3	347.1925	174.0999
1557.7560	1585.7509	793.3791	14	Q	2	260.1605	130.5839
			15	I	1	132.1019	66.5546

MS2 spectrum from apex of 572.95 m/z (3+) peak at 45.8 min in Ca^{2+} replicate 1.
PeptideProphet probability = 0.4740, below the cutoff for identifying spectra.
There are insufficient peaks to localize the phosphosite.
