# Supplementary material for: Plasmodium falciparum Calcium-Dependent Protein Kinase 4 is Critical for Male Gametogenesis and Transmission to the Mosquito Vector
Source: mBio. 2021 Nov 2;12(6):e02575-21. doi: 10.1128/mBio.02575-21 (PMC8561384; doi:10.1128/mBio.02575-21)
Supplement: DATA SET S6 [file mbio.02575-21-sd006.pdf]

Oligo	Forward (5'-3')
PfCDPK4 5'Homo For	T**GCGGCCGC**GTTTCCCTATCTTTTCAGTGCATTTTG
PfCDPK4 5'Homo Rev	TTATGGTTTATTTGATAATGGTTGATACCTTCTTCTTTATATATTGTCTAATG
PfCDPK4 3'Homo For	ATAAAGAAGAAGGTATCAACCATTATCAAATAAACCATAAATCAATTCAATA
PfCDPK4 3'Homo Rev	TAA**GTCGAC**TTAAACTGTGATGATACACATTCTCACTTG
PfCDPK4Guide For	TATTGAAATGAAAGAGAGTAGTGT
PfCDPK4Guide Rev	AAACACACTACTCTCTTTCATTTC
PfCDPK4 Geno5 For	TTTTCTAGGTAAAGTATTAATATATTGTGTGTAAA
PfCDPK4 Geno5 Rev	ACACTACTCTCTTTCATTTCATGTCTCTCA
PfCDPK4 Geno3 For	GAAGTGGATCAAAATAATGATGGAGAA
PfCDPK4 Geno3 Rev	GTATTTAAATATGTGCAGCACAATTTATTC
